# Predicting the survival benefit of liver transplantation in HBV-related acute-on-chronic liver failure: an observational cohort study

**DOI:** 10.1016/j.lanwpc.2022.100638

**Published:** 2022-11-10

**Authors:** Peng Li, Xi Liang, Jinjin Luo, Jiaqi Li, Jiaojiao Xin, Jing Jiang, Dongyan Shi, Yingyan Lu, Hozeifa Mohamed Hassan, Qian Zhou, Shaorui Hao, Huafen Zhang, Tianzhou Wu, Tan Li, Heng Yao, Keke Ren, Beibei Guo, Xingping Zhou, Jiaxian Chen, Lulu He, Hui Yang, Wen Hu, Shiwen Ma, Bingqi Li, Shaoli You, Shaojie Xin, Yu Chen, Jun Li, Chinese Group on the Study of Severe Hepatitis B (COSSH)

**Affiliations:** a State Key Laboratory for Diagnosis and Treatment of Infectious Diseases, National Clinical Research Center for Infectious Diseases, National Medical Center for Infectious Diseases, Collaborative Innovation Center for Diagnosis and Treatment of Infectious Diseases, The First Affiliated Hospital, Zhejiang University School of Medicine, 79 Qingchun Rd., Hangzhou 310003, China; b Precision Medicine Center, Taizhou Central Hospital (Taizhou University Hospital), Taizhou, China; c Key Laboratory of Cancer Prevention and Therapy Combining Traditional Chinese and Western Medicine, Tongde Hospital of Zhejiang Province, Hangzhou, China; d Senior Department of Hepatology, The Fifth Medical Center of Chinese PLA General Hospital, Beijing 100039, China; e Beijing Municipal Key Laboratory of Liver Failure and Artificial Liver Treatment Research, Fourth Department of Liver Disease, Beijing Youan Hospital, Capital Medical University, Beijing 100069, China

**Keywords:** Hepatitis B virus, Acute-on-chronic liver failure, Liver transplantation, Survival benefit, Transplant timing, ACLF, acute-on-chronic liver failure, AUROC, area under the receiver operating characteristic curve, CLIF-C, chronic liver failure Consortium, CLIF-C ACLFs, CLIF-C ACLF score, CLIF-OFs, CLIF-organ failure score, COSSH, Chinese Group on the Study of Severe Hepatitis B, COSSH-ACLFs, COSSH-ACLF score, COSSH-ACLF IIs, COSSH-ACLF II score, EASL, European Association for the Study of the Liver, HBV, hepatitis B virus, HE, hepatic encephalopathy, INR, international normalized ratio, LT, liver transplantation, MELDs, Model for End-stage Liver Disease score, MELD-Nas, MELD-sodium score, PSM, propensity score matching, TB, total bilirubin

## Abstract

**Background:**

Liver transplantation (LT) is an effective therapy for acute-on-chronic liver failure (ACLF) but is limited by organ shortages. We aimed to identify an appropriate score for predicting the survival benefit of LT in HBV-related ACLF patients.

**Methods:**

Hospitalized patients with acute deterioration of HBV-related chronic liver disease (n = 4577) from the Chinese Group on the Study of Severe Hepatitis B (COSSH) open cohort were enrolled to evaluate the performance of five commonly used scores for predicting the prognosis and transplant survival benefit. The survival benefit rate was calculated to reflect the extended rate of the expected lifetime with vs. without LT.

**Findings:**

In total, 368 HBV-ACLF patients received LT. They showed significantly higher 1-year survival than those on the waitlist in both the entire HBV-ACLF cohort (77.2%/52.3%, p < 0.001) and the propensity score matching cohort (77.2%/27.6%, p < 0.001). The area under the receiver operating characteristic curve (AUROC) showed that the COSSH-ACLF II score performed best (AUROC 0.849) at identifying the 1-year risk of death on the waitlist and best (AUROC 0.864) at predicting 1-year outcome post-LT (COSSH-ACLFs/CLIF-C ACLFs/MELDs/MELD-Nas: AUROC 0.835/0.825/0.796/0.781; all p < 0.05). The C-indexes confirmed the high predictive value of COSSH-ACLF IIs. Survival benefit rate analyses showed that patients with COSSH-ACLF IIs 7–10 had a higher 1-year survival benefit rate from LT (39.2%–64.3%) than those with score <7 or >10. These results were prospectively validated.

**Interpretation:**

COSSH-ACLF IIs identified the risk of death on the waitlist and accurately predicted post-LT mortality and survival benefit for HBV-ACLF. Patients with COSSH-ACLF IIs 7–10 derived a higher net survival benefit from LT.

**Funding:**

This study was supported by the 10.13039/501100001809National Natural Science Foundation of China (No. 81830073, No. 81771196) and the National Special Support Program for High-Level Personnel Recruitment (Ten-thousand Talents Program).

**Research in context**

**Evidence before this study**

Acute-on-chronic liver failure (ACLF) is a life-threatening syndrome characterized by multiorgan failure and high short-term mortality rates. Liver transplantation (LT) is an effective therapy but is limited by the shortage of donor organs. Accurately identifying ACLF patients who will have a higher net survival benefit from LT and determining the optimal transplant timing is important to make efficient use of limited donor organs and decrease the risk of futile transplantation. However, the current urgency-based organ allocation system shows lower sensitivity and accuracy for LT in ACLF patients. We searched PubMed for relevant literature published in English with no restrictions on publication date using the terms “liver transplantation OR LT”, “acute-on-chronic liver failure OR ACLF”, “survival benefit” and “predicting”. This process yielded few results, which indicated that very few studies have focused on predicting the survival benefit of LT in ACLF patients, so further studies are urgently needed.

**Added value of this study**

We report a large multicentre cohort of HBV-ACLF patients with longer-term outcomes (1-year) than have been reported before. Moreover, we assess the survival benefit of LT for HBV-ACLF patients for the first time. The findings showed a significant improvement in the 1-year survival probability in the ACLF population who received LT. Compared with four other commonly used scores (COSSH-ACLFs, CLIF-C ACLFs, MELDs and MELD-Nas), COSSH-ACLF IIs showed the best performance in predicting the mortality and survival benefit rate post-LT in HBV-ACLF patients. Patients with COSSH-ACLF IIs 7–10 have a much higher net survival benefit of LT than those with score <7 or >10.

**Implications of all the available evidence**

Our findings can help clinicians determine the appropriate time window of LT and improve the selection of HBV-ACLF patients who will have the best post-LT prognosis in clinical practice.

## Introduction

Hepatitis B virus (HBV) infection is a major global health issue infecting almost one-third of the world's population.^1^, ^2^, ^3^ HBV-related acute-on-chronic liver failure (HBV-ACLF) is a common type of end-stage liver disease in such patients and is characterized by multiorgan failure and high short-term mortality rates.^4^, ^5^, ^6^ Nucleos(t)ide analogue (NA) therapy could effectively suppress HBV replication and reduce the risk of cirrhotic complications and the development of ACLF, but inappropriate cessation of NA is common and associated with the onset and high mortality of HBV-ACLF.^7^, ^8^, ^9^ Liver transplantation (LT) is an effective therapy for ACLF but is limited by a shortage of donor organs.^10^ Studies have demonstrated that only 3%–10% of patients with ACLF receive transplantation.^4^^,^^11^^,^^12^ The main reason for the low transplant rate is that ACLF patients are usually critically unwell, and donor organs may not be available soon enough. The Model for End-stage Liver Disease score (MELDs) and MELD-based organ allocation system, as widely used scores for organ allocation, have indicated lower sensitivity and accuracy for LT in ACLF patients since the impact of extrahepatic organ failure is not reflected in the scores.^13^, ^14^, ^15^

Recently, several definitions and prognostic scores have been developed specifically for ACLF. The European Association for the Study of the Liver (EASL) proposed a definition of ACLF based on acutely decompensated cirrhosis mainly caused by alcohol or hepatitis C virus,^11^ and the Chinese Group on the Study of Severe Hepatitis B (COSSH) proposed a definition for HBV-ACLF regardless of cirrhosis.^4^ The subsequent development of the CLIF-C ACLF score (CLIF-C ACLFs) and the COSH-ACLF score (COSSH-ACLFs) based on the assessment of organ failure have demonstrated a higher predictive performance for patients with ACLF with alcohol- and HBV-related aetiologies, respectively.^4^^,^^16^ A new, simplified version of the COSSH-ACLF score, COSSH-ACLF IIs, based on 6 clinical predictors (total bilirubin (TB), international normalized ratio (INR), age, neutrophils, hepatic encephalopathy (HE) and serum urea) can accurately prognosticate and stratify the short-term mortality of HBV-ACLF patients.^17^ This simplified score shows better predictive ability than the above four scores. However, whether these scoring systems can effectively predict outcomes post-LT and be used to urgently determine the survival benefit rate of transplantation in ACLF patients is still unknown.

Organ allocation systems for LT are usually based on urgency, utility and/or benefit.^18^ Benefit-based organ allocation systems consider both urgency and utility, with a balance between pretransplant and posttransplant outcomes, which may ensure the allocation of the limited donor organs to patients who will have a higher net survival benefit from LT. This system has been evaluated in the context of lung,^19^ kidney^20^ and liver allocation since 2005, and the findings indicate that over 2000 life-years could be saved per year if benefit-based allocation is performed.^21^ The net survival benefit of LT in the HBV-ACLF population has not been fully investigated.

In this study, we enrolled hospitalized patients with acute deterioration of HBV-related chronic liver disease from the COSSH study cohort and evaluated the performance of five commonly used scores in predicting the waitlist and post-LT mortality of HBV-ACLF. Based on the best score, the net survival benefit rates derived from LT were calculated and assessed in HBV-ACLF patients.

## Methods

### Study design

Patients with acute deterioration of HBV-related chronic liver disease were enrolled from the prospectively maintained COSSH study open cohort between January 2015 and December 2020. The predictive performance of HBV-ACLF outcome at days 28, 90, 180, and 365 of five commonly used clinical scores (COSSH-ACLF IIs/COSSH-ACLFs/CLIF-C ACLFs/MELDs/MELD-Nas) was evaluated. The best score was used to draw the survival curve. The survival benefit based on the extra lifespan derived from LT was calculated as the difference in the area under the survival curve between patients with and without LT.^19^ Survival benefit rate analysis was performed to determine the net survival benefit of LT. An external group enrolled from January 2021 to December 2021 was used to prospectively validate the performance of the identified score and the proposed cut-off values for LT.

### Patient screening

**Fig. 1:**
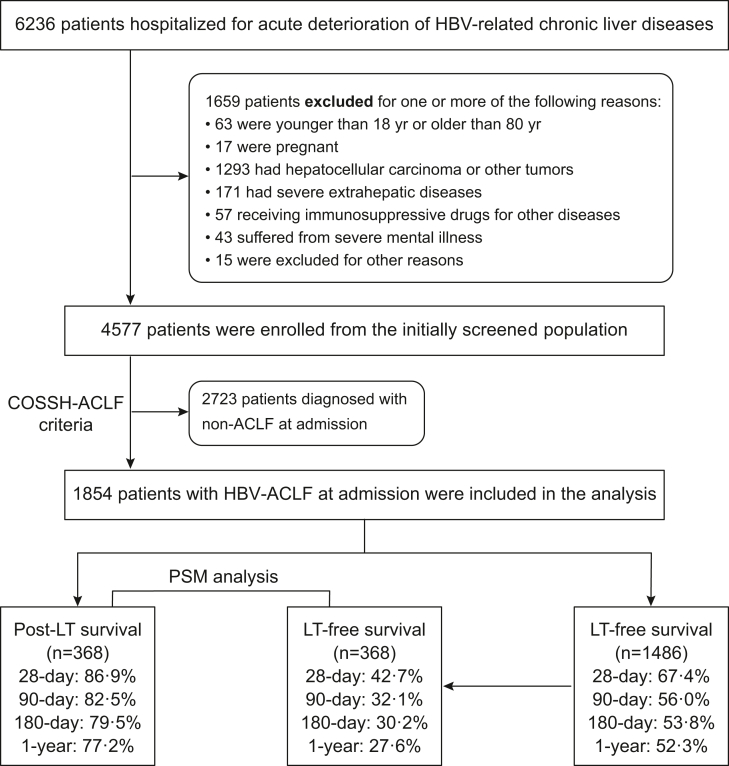
**Patients were screened, enrolled and classified according to the COSSH-ACLF criteria.** ACLF, acute-on-chronic liver failure; COSSH, Chinese Group on the Study of Severe Hepatitis B; HBV-ACLF, hepatitis B virus-related ACLF; LT, liver transplantation; PSM, propensity score matching.

Patients hospitalized for more than 1 day with acute deterioration of HBV-related chronic liver disease were screened and enrolled from the COSSH study open cohort. The exclusion criteria are listed in Fig. 1. After enrolment, according to the COSSH-ACLF criteria, patients were divided into the ACLF group and the non-ACLF group. In the ACLF group, who were included in the final analyses, patients were stratified into 2 groups: 1) the LT group and 2) the non-LT group (Fig. 1).

### Enrolment criteria and COSSH-ACLF definition

The enrolment criteria for HBV-related chronic liver disease were 1) HBV-related acutely decompensated cirrhosis presenting with one or more of the following events: ascites, gastrointestinal bleeding, HE, and infection; and 2) severe liver injury (TB ≥ 5 mg/dL) with previously diagnosed chronic hepatitis B. HBV-ACLF was diagnosed based on the COSSH-ACLF criteria.^4^ This definition identifies HBV-ACLF as a complicated syndrome with a high short-term mortality rate that develops in patients with HBV-related chronic liver disease, regardless of the presence of cirrhosis, and it is characterized by acute deterioration of liver function and hepatic and/or extrahepatic organ failure. HBV-ACLF is classified into 3 grades of severity based on the number of organ failures, namely, ACLF-1, ACLF-2 and ACLF-3. ACLF-1 includes four types of patients: (1) patients with liver failure (serum total bilirubin ≥12 mg/dl) alone with an international normalized ratio (INR) ≥1.5 and/or kidney dysfunction (1.5 mg/dl≤ serum creatinine <2 mg/dl) and/or HE grade I/II under the West-Haven criteria; (2) patients with kidney failure (serum creatinine ≥2 mg/dl) alone; (3) patients with single-organ failure in coagulation (INR ≥2.5) or circulation (use of vasopressors) or the lungs (PaO_2_/FiO_2_ ≤200 or SpO_2_/FiO_2_ ≤214 or use of mechanical ventilation for respiratory failure) and kidney dysfunction and/or HE grade I/II; and (4) patients with cerebral failure (HE grade III/IV) plus kidney dysfunction. ACLF-2 includes patients with 2 organ failures. ACLF-3 includes patients with ≥3 organ failures.

### Data collection

Clinical data were collected at admission or at LT, including demographic data, aetiologies, cirrhosis complications, laboratory indicators, use of antiviral nucleoside analogues for HBV (within six months prior to and during hospitalization), and number and type of organ failures. Patients were followed up for at least 1 year after enrolment. The survival time and status at days 28, 90, 180, and 365 were collected from the electronic data capture system and case report forms.

### Clinical treatment

All participating centres had a regular ward, an intensive care unit and a liver transplantation programme. After admission, all patients received integrative treatment strategies under the clinical guidelines, including rapid restoration of metabolic and haemodynamic stability; provision of nutritional support; agents to protect hepatocytes and promote regeneration; treatment for ascites, infections, HE, and gastrointestinal bleeding; and renal replacement therapy for severe hepatorenal syndrome and plasma exchange for liver dysfunction. Patients were also treated with human serum albumin infusion or plasma transfusion, if needed. All patients with HBV-ACLF in the current study were routinely prescribed NAs, including entecavir, tenofovir disoproxil fumarate or tenofovir alafenamide, for antiviral therapy after admission according to the Consensus Recommendations of the Asian Pacific Association for the Study of the Liver (2014).^22^ The same transplantation administration was used at each study centre.

### Indications for LT

All patients with ACLF in the current study were recommended to be listed for LT according to the EASL Clinical Practice Guidelines for LT.^15^ The degree of medical urgency was determined by the MELD score, and patients with a higher MELD score had a higher priority for transplantation. After integrated treatment, the internist and the surgeon re-evaluated the overall condition of the patient. If the patient had no responses to medical therapies with no sign of getting better and the MELD score was >15 or some exceptions to MELDs still existed, the patient was recommended to be listed for LT on the China Liver Transplant Registry. All donor livers in this study were acquired from donation after brain death (DBD) or cardiac death (DCD), which were all strictly in accordance with the Chinese guidelines on liver donation and the Declaration of Helsinki. The donor-to-recipient arrangements all conformed to the principle of ABO compatibility.

### Propensity score matching (PSM) analysis

To reduce bias and confounding factors, rigorous PSM analysis was performed to assess survival in ACLF patients with and without LT. The model for PSM included the variables age, TB, INR, creatinine, COSSH-ACLF IIs and CLIF-C ACLFs. Matched pairs were selected using the one-to-one nearest-neighbour method with a calliper width of 0.02. Propensity scores were estimated by logistic regression. The R package ‘MatchIt’ was used to match samples.

### Evaluation of five scores for predicting waitlist and post-LT mortality in ACLF patients

The performance of five commonly used scores (COSSH-ACLF IIs/COSSH-ACLFs/CLIF-C ACLFs/MELDs/MELD-Nas) in predicting waitlist and post-LT mortality was compared. The area under the receiver operating characteristic curve (AUROC) and the concordance index were assessed to determine the sensitivity and accuracy of the five scores in predicting waitlist and post-LT mortality at days 28, 90, 180, and 365. Prognostic scores were calculated according to the published formula and are listed as follows: COSSH-ACLF IIs = 1.649 × ln(INR) + 0.457 × HE score (HE grade 0: 1, grade 1-2: 2, grade 3-4: 3) + 0.425 × ln(neutrophil) (10^9^/L) + 0.396 × ln(TB) (μmol/L) + 0.576 × ln(urea) (mmol/L) + 0.033 × age^17^; COSSH-ACLFs = 0.741 × INR + 0.523 × HBV-SOFAs + 0.026 × age + 0.003 × TB(μmol/L)^4^; CLIF-C ACLFs = 10 × [0.33 × CLIF-OFs] + 0.04 × age + 0.63 × ln(white blood cell count) − 2]^16^; MELDs = 3.78 × ln[TB(mg/dL)] + 11.2 × ln(INR) + 9.6 × ln[creatinine (mg/dL)] + 6.43^23^; MELD-Nas = MELDs − Na − (0.025 × MELDs × (140 − Na)) + 140, where the serum sodium concentration was between 125 and 140 mmol/L.^24^ HBV-SOFAs and CLIF-OFs have been reported previously.^25^

### Calculating the survival benefit rate of transplantation

The survival benefit based on transplant urgency and utility was measured by the difference in predicted lifespan with vs. without LT. The predicted lifespan with or without LT was reflected by the area under the survival curve.^19^^,^^21^ The formula used to calculate the survival benefit of transplantation was as follows: for a given candidate, his or her survival benefit (at day 28/90/180/365) = (area under the survival curve with LT − area under the survival curve without LT). To make it comparable at different follow-up times, the survival benefit rate was standardized: (area under the survival curve with LT − area under the survival curve without LT)/time point, where time point = day 28/90/180/365. Considering the dismal prognosis of ACLF, one year was proposed to determine the survival benefit rate of LT.^19^

### Statistical analyses

The results of the measurements are presented as the median (interquartile range) (IQR), mean ± standard deviation, or percentage (%), unless otherwise noted. Continuous variables were compared using Student's t test or the Mann–Whitney U test. The normality of the data distribution was assessed by the Shapiro–Wilk test. Categorical variables were compared using the chi-square test or Fisher's exact test. Survival analysis was carried out using the Kaplan–Meier method, and the log-rank test was used to examine the differences between groups. DeLong's test was used to compare the AUROCs of different scoring systems, and the z score test was used to compare the C-indexes (the R package ‘compareC’). The statistical significance of the survival benefit rate between three different beneficial intervals was calculated using the bootstrap technique with 1000 resamples.^26^ P < 0.05 was considered statistically significant. All statistical analyses were conducted using SPSS software V.25 (SPSS, Chicago, Illinois, USA) and R software, version 4.0 (https://www.r-project.org).

### Ethics approval

The study protocol was approved by the local ethics committee, the Clinical Research Ethics Committee of the First Affiliated Hospital, Zhejiang University School of Medicine (No. 2011-13). All patients were well informed, and written consent was obtained from them or their legal surrogates before enrolment.

### Role of the funding source

The funder of the study had no role in the study design, data collection, analysis, interpretation, or writing of the report.

## Results

### Study population

Among 6236 patients with acute deterioration of HBV-related chronic liver disease, 1659 patients were excluded according to the exclusion criteria, and 4577 patients were included, of whom 1854 patients were diagnosed with ACLF and were finally enrolled in the derivation analysis (Fig. 1). During the study period, a total of 368 patients with ACLF received LT, and 1486 patients remained non-transplanted.

### Clinical characteristics and survival of ACLF patients with and without LT

**Fig. 2:**
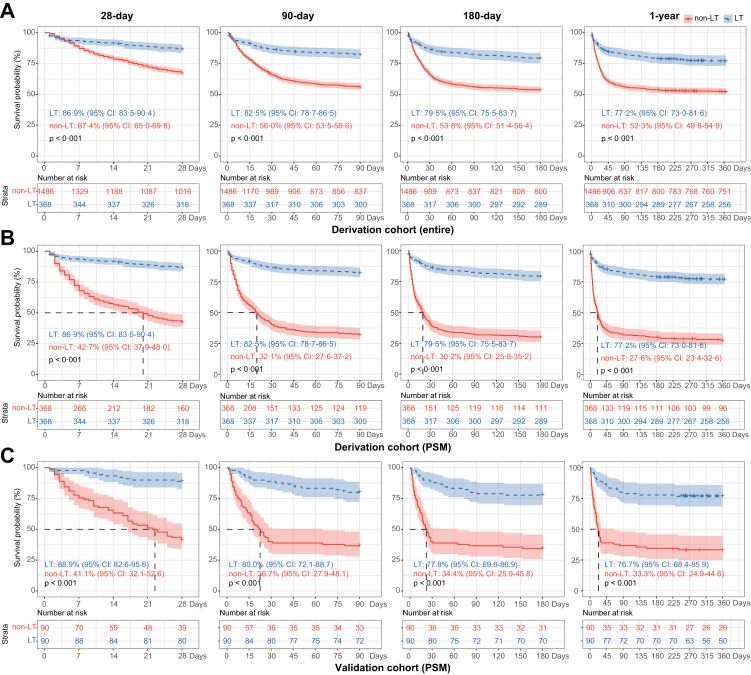
**Kaplan–Meier survival curves of patients with HBV-ACLF stratified by LT at 28 days, 90 days, 180 days and 1 year.** (A) Survival probability of patients with HBV-ACLF in the derivation cohort (entire). (B) Survival probability of patients with HBV-ACLF in the derivation cohort (PSM). (C) Survival probability of patients with HBV-ACLF in the validation cohort (PSM). HBV, hepatitis B virus; ACLF, acute-on-chronic liver failure; LT, liver transplantation; PSM, propensity score matching.

Detailed characteristics of ACLF patients with and without LT are shown in Table 1. In total, 368 ACLF patients underwent LT, and 1486 did not undergo LT. The comprehensive clinical indicators were significantly worse in the LT group than in the non-LT group. The ACLF-LT patients exhibited more frequent complications than the non-LT patients (ascites, 95.7%/66.4%; infections, 47.3%/35.4%; HE, 60.9%/13.2%; hepatorenal syndrome, 21.7%/7.9%; sepsis, 34.0%/13.9%; all p < 0.001; and gastrointestinal bleeding, 6.5%/4.5%, p = 0.092). Except for the frequency of liver failure, the ACLF-LT patients had more frequent organ failure than non-LT patients (coagulation, cerebral, lung, kidney, circulation: 57.6% vs. 29.5%, 35.3% vs. 4.8%, 16.0% vs. 0.7%, 8.7% vs. 5.8%, 4.1% vs. 2.4%, respectively). The ACLF-LT patients were evenly distributed across the ACLF grades (ACLF-1, -2, -3: 32.9%, 33.7%, 33.4%), while non-LT patients were not (ACLF-1, -2, -3: 60.9%, 32.6%, 6.5%). Moreover, the values of the five scores (COSSH-ACLF IIs/COSSH-ACLFs/CLIF-C ACLFs/MELDs/MELD-Nas) were all significantly higher in ACLF-LT patients than in non-LT patients (all p < 0.001), indicating that patients in the ACLF-LT group had more severe disease than those in the ACLF-non-LT group. The survival probabilities in the ACLF-LT group were significantly higher than those in the ACLF-non-LT group (28 days: 86.9% vs. 67.4%; 90 days: 82.5% vs. 56.0%; 180 days: 79.5% vs. 53.8%; 1-year: 77.2% vs. 52.3%, all p < 0.001) (Fig. 2A). To reduce the heterogeneity between these two groups, PSM analysis was performed, yielding 368 pairs (Table 1). After PSM, the survival probabilities in the ACLF-LT group were still much higher than those in the ACLF-non-LT group (p < 0.001) (Fig. 2B). These results indicated that LT significantly improved the survival of ACLF patients.

**Table 1:** Baseline clinical characteristics of ACLF patients with or without LT at baseline in the derivation cohort.

Characteristic	Total (n = 1854)	ACLF-non-LT (Entire cohort) (n = 1486)	ACLF-non-LT (PSM cohort) (n = 368)	ACLF-LT (n = 368)	p^a^	p^b^
Male (no.)	86.7% (1607)	86.8% (1290)	84.8% (312)	86.1% (317)	0.801	0.676
Age (years)	48 [39–56]	48 [40–57]	48 [40–56]	46 [39–54]	0.011	0.113
MAP (mmHg)	86.3 [77.9–95.3]	86.3 [78–95.3]	87.5[78.6–98]	87 [77.6–95]	0.668	0.392
Waiting time to LT (day)	/	/	/	9 [4–21]	N/A	N/A
Antiviral drug use before admission	19.4% (360)	20.8% (306)	18.9% (68)	15.4% (54)	0.029	0.262
Complications						
Gastrointestinal bleeding	4.9% (91)	4.5% (67)	5.4% (20)	6.5% (24)	0.092	0.523
Ascites	72.2% (1339)	66.4% (987)	70.9% (261)	95.7% (352)	<0.001	<0.001
Infection	37.8% (700)	35.4% (526)	47.0% (173)	47.3% (174)	<0.001	1.000
Hepatic encephalopathy	22.7% (420)	13.2% (196)	35.6% (131)	60.9% (224)	<0.001	<0.001
Hepatorenal syndrome	10.7% (198)	7.9% (118)	13.0% (48)	21.7% (80)	<0.001	0.003
Sepsis	17.9% (331)	13.9% (206)	28.8% (106)	34.0% (125)	<0.001	0.132
Virological data						
HBsAg level (IU/mL)	1254 [143–5165]	1238 [143–4690]	974 [111–4893]	1366 [137–7108]	0.238	0.111
HBeAg level (PEIU/ml)	0.34 [0.06–9.38]	0.5 [0.06–9.8]	0.11 [0.06–12.2]	0.2 [0.06–7.0]	0.030	0.050
HBV DNA level (IU/mL)					0.003	0.232
<1000	26.8% (496)	25.2% (375)	28.8% (106)	32.9% (121)	–	–
≥1000	73.2% (1358)	74.8% (1111)	71.2% (262)	67.1% (247)	–	–
Laboratory data						
Albumin (g/L)	31.4 [28.9–34.2]	31.1 [28.6–33.9]	31 [28.8–34]	33.2 [30.2–35.7]	<0.001	<0.001
ALT (U/L)	199 [77–525]	237 [88–580]	271 [87–694]	109 [49–246]	<0.001	<0.001
Total bilirubin (μmol/L)	339 [260–438]	330 [256–428]	359 [270–466]	373 [281–495]	<0.001	0.164
Creatinine (μmol/L)	66 [55–81]	66 [56–79]	69 [57–87]	65 [52–91]	0.547	0.037
Serum urea (mmol/L)	4.4 [3.2–6.6]	4.2 [3.1–6.3]	4.9 [3.5–7.6]	5.6 [3.8-8.3]	<0.001	0.075
Sodium (mmol/L)	138 [135–140]	138 [135–140]	137 [134–139]	138 [134–141]	0.546	0.028
WBC (∗10^9^/L)	6.9 [5.1–9.7]	6.8 [5.0–9.2]	9.3 [6.7–12.7]	8.0 [5.4–11.2]	<0.001	<0.001
Neutrophil (∗10^9^/L)	4.8 [3.3–7.2]	4.6 [3.2–6.7]	6.8 [4.6–10.3]	6.1 [3.8–9.1]	<0.001	0.001
INR	2.1 [1.7-2.7]	2.0 [1.7-2.6]	2.6 [2.1–3.2]	2.6 [2.0–3.3]	<0.001	1.000
Organ failures						
Liver	94.3% (1749)	95.1% (1413)	93.8% (345)	91.3% (336)	0.007	0.262
Kidney	6.4% (118)	5.8% (86)	9.0% (33)	8.7% (32)	0.054	1.000
Coagulation	35.1% (651)	29.5% (439)	61.4% (226)	57.6% (212)	<0.001	0.329
Cerebral	10.8% (201)	4.8% (71)	17.9% (66)	35.3% (130)	<0.001	<0.001
Lungs	3.8% (70)	0.7% (11)	2.2% (8)	16.0% (59)	<0.001	<0.001
Circulation	2.7% (50)	2.4% (35)	7.3% (27)	4.1% (15)	0.100	0.080
ACLF grade					<0.001	<0.001
1	55.3% (1026)	60.9% (905)	29.6% (109)	32.9% (121)	–	–
2	32.8% (609)	32.6% (485)	46.2% (170)	33.7% (124)	–	–
3	11.8% (219)	6.5% (96)	24.2% (89)	33.4% (123)	–	–
Severity scores						
COSSH-ACLF IIs	7.3 [6.6–8.0]	7.1 [6.5–7.8]	7.9 [7.3–8.8]	8.1 [7.3–8.8]	<0.001	0.422
COSSH-ACLFs	6.3 [5.7-7.2]	6.1 [5.6-6.9]	7.0 [6.2–8.0]	7.6 [6.7–8.5]	<0.001	<0.001
CLIF-C ACLFs	42.1 [36.8–48.4]	40.9 [35.9–46.3]	47.8 [42.2–53.3]	48.9 [43.2–55.3]	<0.001	0.092
MELDs	23.4 [20.4–27.4]	23 [20.3–26.6]	26.3 [22.6–30.3]	26.0 [21.6–31.4]	<0.001	0.248
MELD-Nas	24.7 [21.5–28.7]	24.2 [21.3–27.9]	27.6 [24.1–31.9]	27.3 [22.2–32.3]	<0.001	0.164
Survival probability		(LT-free)	(LT-free)	(post-LT)	–	–
28 days	–	67.4%	42.7%	86.9%	<0.001	<0.001
90 days	–	56.0%	32.1%	82.5%	<0.001	<0.001
180 days	–	53.8%	30.2%	79.5%	<0.001	<0.001
1 year	–	52.3%	27.6%	77.2%	<0.001	<0.001

ACLF, acute-on-chronic liver failure; LT, liver transplantation; PSM, propensity score matching; MAP, mean arterial pressure; N/A, not available; ALT, alanine aminotransferase; WBC, white blood cell count; INR, international normalized ratio; COSSH-ACLF IIs, Chinese Group on the Study of Severe Hepatitis B-ACLF II score; COSSH-ACLFs, COSSH-ACLF score; CLIF-C ACLFs, Chronic Liver Failure (CLIF) Consortium ACLF score; MELDs, Model for End-Stage Liver Disease score; MELD-Nas, MELD-sodium score.

Categorical variables are expressed as % (n); continuous variables are expressed as either the mean ± SD or median (IQR).

a Value of comparisons between patients with ACLF-non-LT (Entire cohort) and ACLF-LT.

b Value of comparisons between patients with ACLF-non-LT (PSM cohort) and ACLF-LT.

### Performance of the scores in predicting waitlist and post-LT mortality in ACLF patients

**Fig. 3:**
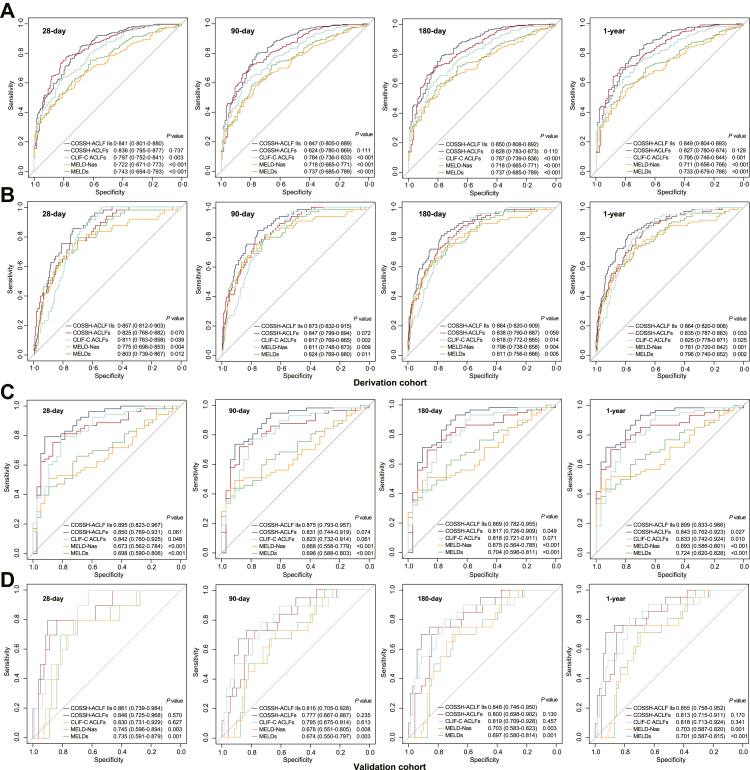
**Receiver operating characteristic (ROC) curves for the performance of the five scores at predicting the waitlist and post-LT mortality of patients with HBV-ACLF at 28 days, 90 days, 180 days and 1 year.** (A) ROC curves for predicting the mortality of HBV-ACLF patients on the waitlist in the derivation cohort. (B) ROC curves for predicting the post-LT mortality of HBV-ACLF patients in the derivation cohort. (C) ROC curves for predicting the mortality of HBV-ACLF patients on the waitlist in the validation cohort. (D) ROC curves for predicting the post-LT mortality of HBV-ACLF patients in the validation cohort. ACLF, acute-on-chronic liver failure; COSSH-ACLF IIs, Chinese Group on the Study of Severe Hepatitis B-ACLF II score; COSSH-ACLFs, COSSH-ACLF score; CLIF-C ACLFs, Chronic Liver Failure (CLIF) Consortium ACLF score; MELD-Nas, Model for End-Stage Liver Disease-sodium score; MELDs, MELD score; LT, liver transplantation.

The ROC curves showed that COSSH-ACLF IIs performed better at identifying the risk of death of ACLF patients on the waitlist than CLIF-C ACLFs, MELDs and MELD-Nas (all p < 0.001) and performed as well as COSSH-ACLFs at days 28, 90, 180, and 365 (all p > 0.05) (Fig. 3A). Moreover, COSSH-ACLF IIs showed significantly higher sensitivity and accuracy in predicting post-LT mortality than CLIF-C ACLFs (28 days: 0.857 vs. 0.811; 90 days: 0.873 vs. 0.817; 180 days: 0.864 vs. 0.818; 1 year: 0.864 vs. 0.825, all p < 0.05), MELDs (28 days: 0.857 vs. 0.803; 90 days: 0.873 vs. 0.824; 180 days: 0.864 vs. 0.811; 1 year: 0.864 vs. 0.796, all p < 0.05) and MELD-Nas (28 days: 0.857 vs. 0.775; 90 days: 0.873 vs. 0.811; 180 days: 0.864 vs. 0.798; 1 year: 0.864 vs. 0.781, all p < 0.05). Compared with COSSH-ACLFs, COSSH-ACLF IIs showed superior predictive ability for 1-year mortality (0.864 vs. 0.835, p = 0.033) and equivalent effectiveness at the other time points (28 days: 0.857 vs. 0.825, p = 0.07; 90 days: 0.873 vs. 0.847, p = 0.072; 180 days: 0.864 vs. 0.838, p = 0.059) (Fig. 3B). We further assessed the discrimination of these scores by the C-index. The C-indexes of COSSH-ACLF IIs for predicting waitlist mortality at days 28, 90, 180, and 365 were the highest among the five scoring systems, mirroring the results of the ROC. COSSH-ACLF IIs also showed the highest C-indexes in predicting post-LT mortality at days 28, 90, 180, and 365 (all p < 0.05) (Supplementary Table S1). These results indicate that COSSH-ACLF IIs can not only identify the risk of death on the waitlist but also accurately predict the post-LT mortality of ACLF patients.

### Survival benefit rate of transplantation in ACLF patients

**Fig. 4:**
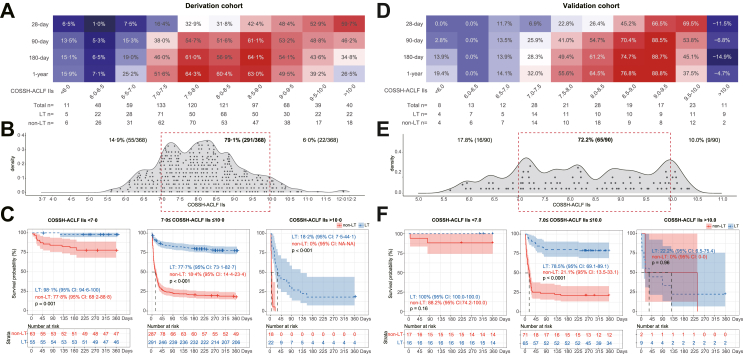
**Survival benefit rate analyses based on COSSH-ACLF IIs.** Survival benefit rate of LT based on COSSH-ACLF IIs at the 28-day, 90-day, 180-day and 1-year follow-ups depicted by a heatmap in (A) the derivation cohort and (D) the validation cohort. The distribution of HBV-ACLF patients who received LT based on COSSH-ACLF IIs in (B) the derivation cohort and (E) the validation cohort. The red dotted box represents the score interval associated with a higher survival benefit rate. The 1-year survival probability of patients with HBV-ACLF with and without LT, stratified into three intervals of COSSH-ACLF IIs (<7, 7–10, >10), in (C) the derivation cohort and (F) the validation cohort. ACLF, acute-on-chronic liver failure; LT, liver transplantation; COSSH-ACLF IIs, Chinese Group on the Study of Severe Hepatitis B-ACLF II score.

As the best score for predicting the mortality of ACLF patients, COSSH-ACLF IIs was used to depict the survival benefit rate at days 28, 90, 180, and 365 (Fig. 4A). The survival benefit rate exhibited a similar trend between score intervals at days 90–365, and a higher benefit rate was limited to the score interval of 7–10 at days 180 and 365. The 1-year survival benefit rate of patients with score 7–10 reached 39.2%–64.3%. For those with score <7, the 1-year survival benefit rate was lower (≤25.2%). At the other end of the spectrum, at score >10, a significant decrease in the 1-year survival benefit rate (≤26.5%) was also observed. The survival benefit rate increased progressively with COSSH-ACLF IIs at 28 days, which may have been influenced by medical support during the perioperative period. To decrease the risk of futile transplantation, patients with COSSH-ACLF IIs 7–10 are proposed as the population who have the highest survival benefit rate (39.2%–64.3%).

The stratification analyses (Fig. 4B) showed that 79.1% of ACLF patients were distributed in the interval of 7–10 (higher survival benefit rate), while 14.9% and 6.0% were in the interval of <7 and >10, respectively. Among these three intervals (COSSH-ACLF IIs <7, 7–10, >10), the comprehensive clinical indicators (TB/INR/creatinine/serum urea/neutrophils/organ failure) deteriorated as the score increased. Patients in the interval of <7 showed a lower frequency of organ failure than those in the interval of 7–10. Patients with score >10 showed a much higher frequency of organ failure, especially of the liver (100%), coagulation (100%), cerebrum (68.2%) and kidney (36.4%) (Table 2). The 1-year survival probability of patients with vs. without LT in each interval was significantly different (COSSH-ACLF IIs <7: 98.1% vs. 77.8%; COSSH-ACLF IIs 7–10: 77.7% vs. 18.4%; and COSSH-ACLF IIs >10: 18.2% vs. 0) (Fig. 4C). The 1-year survival benefit rate in each interval was also significantly different (COSSH-ACLF IIs <7: ≤25.2%; COSSH-ACLF IIs 7–10: 39.2%–64.3%; and COSSH-ACLF IIs >10: ≤26.5%) (Table 2). These results indicate that ACLF patients with COSSH-ACLF IIs of 7–10 have a higher net survival benefit than those with score <7 or >10.

**Table 2:** Clinical characteristics of the ACLF patients who received LT with COSSH-ACLF IIs <7, 7–10 and >10 in the derivation cohort.

Characteristic	COSSH-ACLF IIs <7 (n = 55)	COSSH-ACLF IIs 7–10 (n = 291)	COSSH-ACLF IIs >10 (n = 22)	p^a^	p^b^
Male (no.)	85.5% (47)	85.9% (250)	90.9% (20)	1.000	0.715
Age (years)	39 [35–51]	47 [40–54]	55 [46–60]	0.002	0.005
MAP (mmHg)	86.7 [77.3–93.3]	86.7 [77.5–95.5]	87.5 [78.8–93.9]	0.994	0.752
Waiting time to LT (day)	24 [7–48]	8 [4–18]	7 [3.5–17.5]	<0.001	0.476
Complications before LT					
Gastrointestinal bleeding	12.7% (7)	5.2% (15)	9.5% (2)	0.085	0.792
Ascites	96.3% (53)	96.2% (280)	86.4% (19)	1.000	0.111
Infection	34.5% (19)	47.8% (139)	72.7% (16)	0.076	0.040
Hepatic encephalopathy	32.7% (18)	63.6% (185)	95.5% (21)	<0.001	0.005
Hepatorenal syndrome	7.3% (4)	21.0% (61)	68.2% (15)	0.028	<0.001
Sepsis	20.0% (11)	34.0% (99)	68.2% (15)	0.041	0.001
Virological data					
HBsAg level (IU/mL)	1434 [462–6561]	1250 [112–7249]	2425 [790–16340]	0.838	0.158
HBeAg level (PEIU/ml)	0.1 [0.06–7.0]	0.2 [0.06–7.0]	0.06 [0.05–7.0]	0.658	0.224
HBV DNA level (IU/mL)				0.571	0.449
<1000	29.1% (16)	33.0% (96)	40.9% (9)	–	–
≥1000	70.9% (39)	67.0% (195)	59.1% (13)	–	–
Laboratory data					
Albumin (g/L)	33.9 [30.2–36.3]	33.1 [30.2–35.7]	32.7 [28.6–34.7]	0.537	0.137
Alanine aminotransferase (U/L)	65 [36–182]	109 [54–245]	206 [96–460]	0.043	0.047
Total bilirubin (μmol/L)	275 [191-361]	388 [303–507]	504 [437–627]	<0.001	0.001
Creatinine (μmol/L)	57 [49–77]	64 [51–92]	129 [87–296]	0.039	<0.001
Serum urea (mmol/L)	4.0 [3.0–5.2]	5.8 [4.1–8.3]	10.4 [6.9–22.4]	<0.001	<0.001
Serum sodium (mmol/L)	138 [135–141]	138 [134–141]	137 [132–140]	0.391	0.625
White blood cell (∗10^9^/L)	4.6 [2.9-6.9]	8.3 [5.9–11.5]	10.4 [8.6–12.0]	<0.001	0.016
Neutrophil (∗10^9^/L)	3.0 [2.2–4.6]	6.4 [4.5–9.4]	8.7 [6.5–10.1]	<0.001	0.008
International normalized ratio	1.9 [1.6-2.2]	2.7 [2.1–3.3]	4.6 [3.8-8.1]	<0.001	<0.001
Organ failures					
Liver	74.5% (41)	93.8% (273)	100.0% (22)	<0.001	0.459
Kidney	3.6% (2)	7.6% (22)	36.4% (8)	0.447	<0.001
Coagulation	18.2% (10)	61.9% (180)	100.0% (22)	<0.001	0.001
Cerebral	7.3% (4)	38.1% (111)	68.2% (15)	<0.001	0.010
Lungs	7.3% (4)	16.8% (49)	27.3% (6)	0.021	0.339
Circulation	3.6% (2)	4.1% (12)	4.5% (1)	1.000	1.000
Severity scores					
COSSH-ACLF IIs	6.5 [6.3–6.8]	8.2 [7.5–8.8]	10.3 [10.1–10.7]	<0.001	<0.001
COSSH-ACLFs	6.0 [5.7-6.4]	7.7 [7.0–8.5]	10.5 [10.0–12.2]	<0.001	<0.001
CLIF-C ACLFs	37.3 [33.5–41.2]	50.1 [45.1–55.4]	62.8 [59.3–64.4]	<0.001	<0.001
MELDs	20.4 [17.7–22.0]	26.5 [22.3–31.2]	41.1 [39.2–45.1]	<0.001	<0.001
MELD-Nas	21.3 [17.6–24.8]	27.6 [23.3–31.8]	41.1 [39.3–45.5]	<0.001	<0.001
Survival benefit rate					
1 year	≤25.2%	39.2%-64.3%	≤26.5%	<0.001^c^	<0.001^c^

ACLF, acute-on-chronic liver failure; LT, liver transplantation; MAP, mean arterial pressure; COSSH-ACLF IIs, Chinese Group on the Study of Severe Hepatitis B-ACLF II score; COSSH-ACLFs, COSSH-ACLF score; CLIF-C ACLFs, Chronic Liver Failure Consortium ACLF score; MELDs, Model for End-Stage Liver Disease score; MELD-Nas, MELD-sodium score.

Categorical variables are expressed as % (n); continuous variables are expressed as either the mean ± SD or median (IQR).

a Value of comparisons between patients with COSSH-ACLF IIs <7 and 7–10.

b Value of comparisons between patients with COSSH-ACLF IIs >10 and 7–10.

c Is calculated using the bootstrap technique with 1000 resamples.

### External validation of the results

To validate the performance of COSSH-ACLF IIs and the proposed cut-off values for LT, an external cohort of 180 patients with HBV-ACLF (90 patients received LT and 90 patients remained on the waitlist) was prospectively enrolled (Supplementary Tables S2 and S3). The survival probabilities in the ACLF-LT group were significantly higher than those in the ACLF-non-LT group (28 days: 88.9% vs. 41.1%, 90 days: 80.0% vs. 36.7%, 180 days: 77.8% vs. 34.4%, 1 year: 76.7% vs. 33.3%, all p < 0.001) (Fig. 2C). The AUROC showed that COSSH-ACLF IIs performed better at identifying the 1-year risk of death of ACLF patients on the waitlist (0.899) than COSSH-ACLFs (0.843, p = 0.027), CLIF-C ACLFs (0.833, p = 0.010), MELDs (0.724, p < 0.001) and MELD-Nas (0.693, p < 0.001). At other time points (28, 90, 180 days), COSSH-ACLF IIs also showed equivalent or better predictive abilities (Fig. 3C). For predicting post-LT mortality, COSSH-ACLF IIs performed equivalently at days 28, 90, 180, and 365 (AUROC: 0.861, 0.816, 0.848, 0.855) compared to COSSH-ACLFs (0.846, 0.777, 0.800, 0.813, all p > 0.05) and CLIF-C ACLFs (0.830, 0.795, 0.819, 0.818, all p > 0.05) and significantly higher values compared to those of the MELDs (0.735, 0.674, 0.697, 0.701, all p < 0.001) and MELD-Nas (0.745, 0.678, 0.703, 0.703, all p < 0.001) (Fig. 3D). The C-index analysis confirmed the predictive ability of COSSH-ACLF IIs in the validation cohort (Supplementary Table S4). The survival benefit rate analysis exhibited similar trends between score intervals to those in the derivation cohort, and the higher survival benefit rate was also limited to the score interval of 7–10 at 1 year (Fig. 4D). Moreover, the results of the distribution (Fig. 4E) and survival curve (Fig. 4F) of patients in the three intervals (<7, 7–10, >10) were all similar to those in the derivation cohort. These results prospectively validate the robustness of the performance of COSSH-ACLF IIs and the proposed cut-off values for deciding upon LT.

## Discussion

Identifying an appropriate score for predicting the post-LT prognosis and the net survival benefit of LT in ACLF patients is important to decrease the risk of futile transplantation in the face of donor organ shortages.^10^^,^^27^ In this multicentre observational cohort study, we observed a significant improvement in the 1-year survival probability post-LT in the HBV-ACLF population. COSSH-ACLF IIs, which exhibited the best performance in predicting both waitlist and post-LT mortality in HBV-ACLF patients, showed a high-transplant-survival-benefit interval of 7–10. These findings may help clinicians determine the optimal timing of LT in HBV-ACLF patients and improve the transplant efficiency.

The current organ allocation system is mainly based on three principles: urgency, utility and benefit.^18^ Under urgency-based systems, candidates with worse outcomes are given higher priority for LT, while utility-based systems assign priority in terms of expected outcomes post-LT. Benefit-based organ allocation balanced with urgency and utility has also been evaluated in prioritizing LT. A recent study demonstrated that patients with a lower risk of death before LT showed no survival benefit post-LT, so determining the survival benefit rate to identify patients who would benefit most from LT should be considered a policy priority.^28^ A subsequent study created and evaluated a survival benefit score for patients with chronic liver failure based on the 5-year lifetime post-LT and patient and donor characteristics. The results indicated that >2000 life-years could be saved per year if a benefit-based allocation system was implemented.^21^ Few studies have focused on the survival benefit rate of ACLF patients from LT, especially in HBV-ACLF, which is a complicated syndrome with high short-term mortality, and LT is always urgently considered but disadvantaged by the current organ allocation system and limited by donor organ shortages.^29^ Other ACLF is a dynamic and reversible disease, and some ACLF grade 1 patients with and without LT have a similarly high survival probability,^30^^,^^31^ which indicates the potential for unnecessary LT and further increases the shortage of donor organs. In our cohort, we systemically evaluated 368 LT patients with HBV-ACLF. The results showed that a significant improvement in survival probability post-LT was observed in the entire cohort. Given the heterogeneity and different disease severities between the ACLF-LT and ACLF-non-LT groups, PSM analysis was further performed. We tried to match the disease severity with individual clinical characteristics directly, but it was hard to match some variables, such as the frequency of ascites, HE and lung failure. COSSH-ACLF IIs and the CLIF-C ACLFs are two widely used scores that can accurately and sensitively reflect the disease severity of ACLF patients, and patients with the same score are considered to have the same disease severity.^16^^,^^17^^,^^32^ We included these scores as variables in deriving the propensity score. The results in the PSM cohort also showed a significant improvement in survival in patients with LT. Even so, the detailed survival benefit rate of LT in ACLF patients should be further clarified.

To clarify the survival benefit rate, an appropriate prognostic score with high performance in predicting outcomes is necessary. Many studies have indicated that MELDs- or MELD-based (including MELD-Nas and ‘exceptions’ to MELDs) allocation systems show lower sensitivity and accuracy for LT in ACLF patients, as they cannot reflect the impact of cirrhosis complications and extrahepatic organ failure.^10^^,^^15^^,^^29^ Recently, the organ failure-based CLIF-C ACLFs and COSSH-ACLFs were developed, showing better predictive performance for LT-free mortality in alcohol- and HBV-related ACLF patients.^4^^,^^16^ However, these two scores are based on complicated scales of six types of organ failure with eleven clinical predictors. The newly simplified version, COSSH-ACLF IIs, based on six predictors (TB/INR/age/neutrophils/HE/serum urea), further improved the prognostic ability for patients with HBV-ACLF. This new score can easily divide patients into three different strata with significantly different mortality rates. COSSH-ACLF IIs also includes organ failure predictors for the liver, coagulation, cerebrum and kidney.^17^ In this study, we evaluated five scores for predicting waitlist and post-LT mortality in ACLF patients. The ROC curves and the C-indexes both showed that COSSH-ACLF IIs performed better at days 28, 90, 180, and 365 than COSSH-ACLFs, CLIF-C ACLFs, MELDs and MELD-Nas, especially at 1 year (all p < 0.05). Although the significant differences between COSSH-ACLF IIs and COSSH-ACLFs were small and the 95% confidence intervals for these two measures overlapped, COSSH-ACLF IIs still showed superiority in its simplified calculation process with fewer indicators and its ease of use in clinical practice. These results were consistent with those of a previous study showing that COSSH-ACLF IIs had the highest sensitivity and accuracy in predicting LT-free mortality in HBV-ACLF patients.^17^^,^^32^ Further analyses with COSSH-ACLF IIs showed an interval distribution of the survival benefit rate at 1 year post-LT. Patients with score 7–10 had a significantly higher survival benefit rate (39.2%–64.3%), while patients with score <7 (≤25.2%) or >10 (≤26.5%) showed a lower benefit rate. These results indicate a potentially futile LT in mild ACLF patients with COSSH-ACLF IIs <7 and in very sick ACLF patients with COSSH-ACLF IIs >10. COSSH-ACLF IIs is the best score for predicting the 1-year outcome and the survival benefit rate post-LT in ACLF patients.

The optimal timing for LT remains complicated since ACLF can reverse or worsen in a few days.^32^ Accurate stratification based on the survival benefit rate can help to determine the transplant timing for ACLF patients. In this study, COSSH-ACLF IIs was the best score, so we used it to evaluate the survival benefit rate post-LT and identified three different benefit intervals (COSSH-ACLF IIs <7, 7–10, >10). Patients with score 7–10 had a higher 1-year survival benefit rate (39.2%–64.3%), while score <7 or >10 exhibited a lower 1-year survival benefit rate (≤25.2% or ≤26.5%). Patients with score <7 had fewer complications of infection, HE and hepatorenal syndrome and lower frequencies of all 6 types of organ failure than those with score 7–10. Such patients also had higher 1-year survival probability without LT (77.8%), which indicated a high potential reversibility of the disease and a lower transplant urgency in such patients. The patients with score >10 exhibited more complications of infection, HE and hepatorenal syndrome and much higher frequencies of all 6 types of organ failure, which indicated a very sick status, so such patients would usually be considered for urgent LT since they had a 100% 1-year mortality rate on the waitlist. However, severe complications and multiorgan failure lead to significantly worse outcomes post-LT. As our results showed, such patients who received LT had only an 18.2% 1-year survival probability and a ≤26.5% net survival benefit rate, which indicated that such patients may be too sick to be transplanted, and the transplant timing should be moved forward. Considering the severe shortage of donor organs, we propose patients with COSSH-ACLF IIs of 7–10 as the population who have a higher net survival benefit. This may help clinicians determine the appropriate transplant timing and improve the selection of patients who will have the best post-LT prognosis in clinical practice.

Our study had some limitations. Its retrospective nature might bias the results. Considering the dismal prognosis of ACLF, we used the 1-year mortality and survival benefit as the primary outcomes; however, the transplant benefit is usually studied in the long term, so a follow-up time of more than 1 year will be necessary to assess the transplant survival benefit of ACLF in the future. Few LT patients had COSSH-ACLF IIs >10; to some extent, this may impact the results, and this should be overcome with larger cohorts. Only HBV-infected patients were enrolled in this study, which might limit the generalization of our conclusions to other aetiologies, such as alcoholic liver disease; however, the results remain relevant because of the major issue of HBV infection worldwide.

In summary, we report a large multicentre cohort of HBV-ACLF patients and provide a longer-term outcome (1 year) of HBV-ACLF than has been reported. We also identify a simplified prognostic score with high accuracy and sensitivity for HBV-ACLF to assess post-LT prognosis and the appropriate timing for LT based on the survival benefit rate. Our cohort was prospectively maintained, and the results from the prospective cohort verify the quality of the data. Our results could help clinicians determine the time window of LT and improve the selection of HBV-ACLF patients who will have the best post-LT prognosis in clinical practice.

## Contributors

P.L., X.L., J.L., J.L. and J.X. contributed equally to this work. J.L. designed the study and obtained financial support for this study. J.L. and Y.C. supervised the study. P.L., J.L., J.L., Q.Z., H.Z., T.W., T.L., K.R., B.G., X.Z., J.C., L.H., H.Y., W.H., S.M., B.L., S.Y., S.X. and Y.C. collected the data, and P.L., X.L., J.L., J.L. and J.X. verified the data. X.L. and P.L. performed the statistical analyses, and J.L., P.L., X.L., J.L., J.X., J.J., D.S., Y.L., H.H. and S.H. interpreted the results. J.L., P.L. and X.L. wrote the manuscript. All authors were involved in the critical revision of the manuscript and approved the final version.

## Data sharing statement

Anonymised individual-level data and datasets generated or analysed during the current study are available for researchers who provide a methodologically sound proposal. Proposals should be directed to Prof. Jun Li (lijun2009@zju.edu.cn). The data will be available beginning 3 months after publication of this article, with no end date.

## Declaration of interests

None of the authors have competing interests to declare.
