# Supplementary material for: Predicting the survival benefit of liver transplantation in HBV-related acute-on-chronic liver failure: an observational cohort study
Source: Lancet Reg Health West Pac. 2022 Nov 10;32:100638. doi: 10.1016/j.lanwpc.2022.100638 (PMC9923183; doi:10.1016/j.lanwpc.2022.100638)
Supplement: Supplementary Materials [file mmc1.docx]

**SUPPLEMENTARY MATERIALS**

**Predicting the survival benefit of liver transplantation in HBV-related acute-on-chronic liver failure: An observational cohort study**

Peng Li, Xi Liang, Jinjin Luo, Jiaqi Li, Jiaojiao Xin, Jing Jiang, Dongyan Shi, Yingyan Lu, Hozeifa Mohamed Hassan, Qian Zhou, Shaorui Hao, Huafen Zhang, Tianzhou Wu, Tan Li, Heng Yao, Keke Ren, Beibei Guo, Xingping Zhou, Jiaxian Chen, Lulu He, Hui Yang, Wen Hu, Shiwen Ma, Bingqi Li, Shaoli You, Shaojie Xin, Yu Chen, Jun Li, on behalf of the Chinese Group on the Study of Severe Hepatitis B (COSSH).

**Contents**

- Supplementary Table 1
- Supplementary Table 2
- Supplementary Table 3
- Supplementary Table 4

**Supplementary Table 1. The C-indexes of five scores for predicting the waitlist and post-LT mortality of patients with HBV-ACLF at days 28, 90, 180 and 1 year in the derivation cohort.**

|  | COSSH-ACLF II score  C-index (95% CI) | COSSH-ACLF score  C-index (95% CI) | CLIF-C ACLF score  C-index (95% CI) | MELD score  C-index (95% CI) | MELD-Na score  C-index (95% CI) |
| --- | --- | --- | --- | --- | --- |
| Waitlist |  |  |  |  |  |
| 28 days  P value | 0·814 (0·796-0·833)  ·· | 0·808 (0·789-0·827)  0·104 | 0·745 (0·723-0·767)  <0·001 | 0·732 (0·709-0·756)  <0·001 | 0·727 (0·703-0·750)  <0·001 |
| 90 days  P value | 0·802 (0·786-0·818)  ·· | 0·793 (0·776-0·810)  0·019 | 0·722 (0·703-0·742)  <0·001 | 0·721 (0·701-0·741)  <0·001 | 0·717 (0·697-0·737)  <0·001 |
| 180 days  P value | 0·798 (0·782-0·814)  ·· | 0·788 (0·772-0·805)  0·014 | 0·717 (0·698-0·736)  <0·001 | 0·715 (0·695-0·735)  <0·001 | 0·712 (0·692-0·732)  <0·001 |
| 1 year  P value | 0·797 (0·781-0·812)  ·· | 0·787 (0·770-0·803)  0·011 | 0·715 (0·696-0·734)  <0·001 | 0·628 (0·603-0·653)  <0·001 | 0·711 (0·691-0·730)  <0·001 |
| Post-LT |  |  |  |  |  |
| 28 days  P value | 0·833 (0·791-0·874)  ·· | 0·806 (0·756-0·855)  0·013 | 0·795 (0·750-0·839)  0·001 | 0·781 (0·724-0·840)  0·006 | 0·755 (0·683-0·827)  0·002 |
| 90 days  P value | 0·837 (0·801-0·874)  ·· | 0·817 (0·775-0·859)  0·021 | 0·840 (0·749-0·833)  <0·001 | 0·792 (0·743-0·841)  0·004 | 0·778 (0·720-0·836)  0·003 |
| 180 days  P value | 0·826 (0·788-0·864)  ·· | 0·807 (0·765-0·848)  0·028 | 0·787 (0·748-0·827)  <0·001 | 0·779 (0·731-0·826)  0·002 | 0·766 (0·712-0·820)  0·002 |
| 1 year  P value | 0·825 (0·789-0·861)  ·· | 0·801 (0·760-0·842)  0·007 | 0·786 (0·747-0·825)  <0·001 | 0·766 (0·720-0·813)  <0·001 | 0·752 (0·699-0·804)  <0·001 |

P value of comparisons between COSSH-ACLF II score and the other scores. (z score test). LT, liver transplantation; HBV-ACLF, hepatitis B virus-related acute-on-chronic liver failure; COSSH-ACLF II score, Chinese Group on the Study of Severe Hepatitis B-ACLF II score; CLIF-C ACLF score, Chronic Liver Failure (CLIF)-Consortium ACLF score; MELD score, Model for End-Stage Liver Disease score; MELD-Na score, MELD-sodium score.

**Supplementary Table 2. Clinical characteristics of the ACLF-LT patients in the derivation and validation cohorts.**

| Characteristics | Derivation  (n=368) | Validation  (n=90) | p value |  |
| --- | --- | --- | --- | --- |
| Male (no.) | 86·1% (317) | 86·7% (78) | 1·000 |  |
| Age (years) | 46 [39-54] | 50±12 | 0·004 |  |
| MAP (mmHg) | 87 [77·6-95] | 85·0 [80·5, 91·5] | 0·354 |  |
| Waiting time to LT (day) | 9 [4-21] | 7 [2-13] | 0·036 |  |
| Antiviral drug use before admission | 66·0% (243) | 40·0% (36) | <0·001 |  |
| Complications before LT |  |  |  |  |
| Gastrointestinal bleeding | 6·5% (24) | 20·0% (18) | <0·001 |  |
| Ascites | 95·7% (352) | 93·3% (84) | 0·518 |  |
| Bacterial infection | 47·3% (174) | 36·7% (33) | 0·090 |  |
| Hepatic encephalopathy | 60·9% (224) | 63·3% (57) | 0·757 |  |
| Hepatorenal syndrome | 21·7% (80) | 27·8% (25) | 0·279 |  |
| Sepsis | 34·0% (125) | 26·7% (24) | 0·186 |  |
| Virological data |  |  |  |  |
| HBsAg level (IU/mL) | 1366 [137-7108] | 363 [15, 2821] | 0·001 |  |
| HBeAg level (PEIU/ml) | 0·2 [0·06-7·0] | 0·08 [0·06, 0·38] | 0·930 |  |
| HBV DNA level (IU/mL) |  |  | 0·005 |  |
| < 1000 | 32·9% (121) | 48·9% (44) | ·· |  |
| ≥ 1000 | 67·1% (247) | 51·1% (46) | ·· |  |
| Laboratory indicators |  |  |  |  |
| Albumin (g/L) | 33·2 [30·2-35·7] | 33·7 [30·7, 36·7] | 0·070 |  |
| Alanine aminotransferase (U/L) | 109 [49-246] | 119 [50, 296] | 0·534 |  |
| Total bilirubin (μmol/L) | 373 [281-495] | 365 [234, 485] | 0·064 |  |
| Creatinine (μmol/L) | 65 [52-91] | 87 [57, 151] | 0·118 |  |
| Serum urea (mmol/L) | 5·6 [3·8-8·3] | 6·3 [3·9, 12·4] | 0·981 |  |
| Serum sodium (mmol/L) | 138 [134-141] | 138 [136, 141] | 0·120 |  |
| White blood cell (*10^9^/L) | 8·0 [5·4-11·2] | 7·5 [3·9, 10·8] | 0·009 |  |
| Neutrophil (*10^9^/L) | 6·1 [3·8-9·1] | 5·5 [2·5, 8·9] | 0·311 |  |
| International normalized ratio | 2·6 [2·0-3·3] | 2·6 [1·8, 3·6] | 0·309 |  |
| Organ failures |  |  |  | |
| Liver | 91·3% (336) | 95·6% (86) | 0·261 |  |
| Kidney | 8·7% (32) | 22·2% (20) | 0·001 |  |
| Coagulation | 57·6% (212) | 54·4% (49) | 0·671 |  |
| Cerebral | 35·3% (130) | 35·6% (32) | 1·000 |  |
| Lungs | 16·0% (59) | 20·0% (18) | 0·456 |  |
| Circulation | 4·1% (15) | 10·0% (9) | 0·046 |  |
| ACLF grade |  |  | 0·292 | |
| 1 | 32·9% (121) | 25·6% (23) | ·· | |
| 2 | 33·7% (124) | 33·3% (30) | ·· | |
| 3 | 33·4% (123) | 41·1% (37) | ·· | |
| Severity scores |  |  |  | |
| COSSH-ACLF IIs | 8·1 [7·3-8·8] | 8·2 [7·2, 9·4] | 0·453 |  |
| COSSH-ACLFs | 7·6 [6·7-8·5] | 8·0 [6·4, 9·0] | 0·832 |  |
| CLIF-C ACLFs | 48·9 [43·2-55·3] | 48·7 [41·2, 58·5] | 0·490 |  |
| MELDs | 26·0 [21·6-31·4] | 24·2 [19·8, 30·0] | 0·024 |  |
| MELD-Nas | 27·3 [22·2-32·3] | 25·6 [20·5, 29·8] | 0·026 |  |
| Survival probability | (LT-free) | (post-LT) |  |  |
| 28-day | 86·9% | 88·9% | 0·570 |  |
| 90-day | 82·5% | 80·0% | 0·230 |  |
| 180-day | 79·5% | 77·8% | 0·370 |  |
| 1-year | 77·2% | 76·7% | 0·160 |  |

Categorical variables are expressed as % (n); continuous variables are expressed as either the mean ± SD or median (IQR).

ACLF, acute-on-chronic liver failure; LT, liver transplantation; MAP, mean arterial pressure; COSSH-ACLF IIs, Chinese Group on the Study of Severe Hepatitis B-ACLF II score; COSSH-ACLFs, COSSH-ACLF score; CLIF-C ACLFs, Chronic Liver Failure (CLIF) Consortium ACLF score; MELDs, Model for End-Stage Liver Disease score; MELD-Nas, MELD-sodium score.

**Supplementary Table 3. Clinical characteristics of the ACLF patients with and without LT at baseline in the validation cohort.**

| Characteristics | Total  (n=180) | ACLF-non-LT  (n=90) | ACLF-LT  (n=90) | p^*^ value |
| --- | --- | --- | --- | --- |
| Male (no.) | 86·1% (155) | 85·6% (77) | 86·7% (78) | 1·000 |
| Age (years) | 51±12 | 51±13 | 50±12 | 0·634 |
| MAP (mmHg) | 84·7 [79·2, 93·3] | 84·7 [78·1, 95·0] | 85·0 [80·5, 91·5] | 0·838 |
| Waiting time to LT (day) | ·· | ·· | 7 [2-13] | N/A |
| Antiviral drug use before admission | 35·0% (63) | 30·0% (27) | 40·0% (36) | 0·161 |
| Complications |  |  |  |  |
| Gastrointestinal bleeding | 15·0% (27) | 10·0% (9) | 20·0% (18) | 0·095 |
| Ascites | 79·4% (143) | 65·6% (59) | 93·3% (84) | <0·001 |
| Infection | 39·4% (71) | 42·2% (38) | 36·7% (33) | 0·542 |
| Hepatic encephalopathy | 46·7% (84) | 30·0% (27) | 63·3% (57) | <0·001 |
| Hepatorenal syndrome | 22·8% (41) | 17·8% (16) | 27·8% (25) | 0·155 |
| Sepsis | 23·3% (42) | 20·0% (18) | 26·7% (24) | 0·292 |
| Virological data |  |  |  |  |
| HBsAg level (IU/mL) | 543 [48, 2588] | 702 [113, 2557] | 363 [15, 2821] | 0·130 |
| HBeAg level (PEIU/ml) | 0·08 [0·06, 0·34] | 0·07 [0·06, 0·17] | 0·08 [0·06, 0·38] | 0·192 |
| HBV DNA level (IU/mL) |  |  |  | 0·049 |
| < 1000 | 36·7%% (66) | 24·4% (22) | 48·9% (44) | ·· |
| ≥ 1000 | 63·3% (114) | 75·6% (68) | 51·1% (46) | ·· |
| Laboratory data |  |  |  |  |
| Albumin (g/L) | 32·2 [29·5, 34·7] | 30·8 [28·7, 32·6] | 33·7 [30·7, 36·7] | <0·001 |
| ALT (U/L) | 131 [70, 334] | 141 [84, 359] | 119 [50, 296] | 0·109 |
| Total bilirubin (μmol/L) | 370 [280, 474] | 375 [299, 472] | 365 [234, 485] | 0·356 |
| Creatinine (μmol/L) | 77 [58, 128] | 70 [58, 95] | 87 [57, 151] | 0·099 |
| Serum urea (mmol/L) | 5·8 [3·7, 10·7] | 5·7 [3·6, 9·2] | 6·3 [3·9, 12·4] | 0·361 |
| Sodium (mmol/L) | 138 [135, 141] | 138 [135, 140] | 138 [136, 141] | 0·305 |
| WBC (*10^9^/L) | 7·6 [4·9, 11·1] | 7·6 [5·9, 11·3] | 7·5 [3·9, 10·8] | 0·164 |
| Neutrophil (*10^9^/L) | 5·6 [3·2, 8·9] | 5·7 [3·9, 9·0] | 5·5 [2·5, 8·9] | 0·324 |
| INR | 2·5 [1·9, 3·2] | 2·4 [2·0, 3·2] | 2·6 [1·8, 3·6] | 0·877 |
| Organ failures |  |  |  |  |
| Liver | 96·7% (174) | 97·8% (88) | 95·6% (86) | 0·678 |
| Kidney | 18·3% (33) | 14·4% (13) | 22·2% (20) | 0·248 |
| Coagulation | 52·2% (94) | 50·0% (45) | 54·4% (49) | 0·654 |
| Cerebral | 27·8% (50) | 20·0% (18) | 35·6% (32) | 0·031 |
| Lungs | 13·3% (24) | 6·7% (6) | 20·0% (18) | 0·016 |
| Circulation | 10·0% (18) | 10·0% (9) | 10·0% (9) | 1·000 |
| ACLF grade |  |  |  | 0·054 |
| 1 | 30·6% (55) | 35·6% (32) | 25·6% (23) | ·· |
| 2 | 36·7% (66) | 40·0% (36) | 33·3% (30) | ·· |
| 3 | 32·8% (59) | 24·4% (22) | 41·1% (37) | ·· |
| Severity scores |  |  |  |  |
| COSSH-ACLF IIs | 8·2 [7·2, 9·2] | 8·2 [7·2, 9·0] | 8·2 [7·2, 9·4] | 0·462 |
| COSSH-ACLFs | 7·3 [6·2, 8·8] | 7·0 [6·1, 8·4] | 8·0 [6·4, 9·0] | 0·107 |
| CLIF-C ACLFs | 48·4 [41·2, 56·7] | 48·1 [41·8, 54·5] | 48·7 [41·2, 58·5] | 0·334 |
| MELDs | 23·5 [19·4, 29·5] | 23·1 [19·4, 28·9] | 24·2 [19·8, 30·0] | 0·597 |
| MELD-Nas | 24·5 [19·9, 29·5] | 24·0 [19·9, 28·8] | 25·6 [20·5, 29·8] | 0·558 |
| Survival probability |  | (LT-free) | (post-LT) |  |
| 28-day | ·· | 41·1% | 88·9% | <0·001 |
| 90-day | ·· | 36·7% | 80·0% | <0·001 |
| 180-day | ·· | 34·4% | 77·8% | <0·001 |
| 1-year | ·· | 33·3% | 76·7% | <0·001 |

Categorical variables are expressed as % (n); continuous variables are expressed as either the mean ± SD or median (IQR).

P^*^ value of comparisons between patients with ACLF-non-LT and ACLF-LT.

ACLF, acute-on-chronic liver failure; LT, liver transplantation; MAP, mean arterial pressure; N/A, not available; ALT, alanine aminotransferase; WBC, white blood cell count; INR, international normalized ratio; COSSH-ACLF IIs, Chinese Group on the Study of Severe Hepatitis B-ACLF II score; COSSH-ACLFs, COSSH-ACLF score; CLIF-C ACLFs, Chronic Liver Failure (CLIF) Consortium ACLF score; MELDs, Model for End-Stage Liver Disease score; MELD-Nas, MELD-sodium score.

**Supplementary Table 4. The C-indexes of five scores for predicting the waitlist and post-LT mortality of patients with HBV-ACLF at days 28, 90, 180 and 1 year in the validation cohort.**

|  | COSSH-ACLF II score  C-index (95% CI) | COSSH-ACLF score  C-index (95% CI) | CLIF-C ACLF score  C-index (95% CI) | MELD score  C-index (95% CI) | MELD-Na score  C-index (95% CI) |
| --- | --- | --- | --- | --- | --- |
| Waitlist |  |  |  |  |  |
| 28 days  P value | 0·819 (0·763-0·874)  ·· | 0·784 (0·725-0·844)  0·007 | 0·770 (0·713-0·827)  <0·001 | 0·672 (0·602-0·742)  <0·001 | 0·656 (0·579-0·733)  <0·001 |
| 90 days  P value | 0·810 (0·755-0·864)  ·· | 0·772 (0·716-0·829)  0·003 | 0·761 (0·705-0·816)  <0·001 | 0·659 (0·594-0·725)  <0·001 | 0·642 (0·570-0·715)  <0·001 |
| 180 days  P value | 0·808 (0·753-0·862)  ·· | 0·767 (0·712-0·823)  0·002 | 0·759 (0·703-0·815)  <0·001 | 0·660 (0·594-0·725)  <0·001 | 0·643 (0·571-0·715)  <0·001 |
| 1 year  P value | 0·809 (0·757-0·862)  ·· | 0·769 (0·715-0·824)  0·001 | 0·760 (0·705-0·815)  <0·001 | 0·663 (0·599-0·727)  <0·001 | 0·646 (0·575-0·717)  <0·001 |
| Post-LT |  |  |  |  |  |
| 28 days  P value | 0·852 (0·744-0·960)  ·· | 0·839 (0·732-0·946)  0·304 | 0·817 (0·730-0·903)  0·117 | 0·717 (0·599-0·836)  0·001 | 0·728 (0·604-0·852)  0·005 |
| 90 days  P value | 0·807 (0·711-0·904)  ·· | 0·771 (0·675-0·867)  0·054 | 0·774 (0·679-0·868)  0·057 | 0·661 (0·561-0·762)  <0·001 | 0·667 (0·562-0·771)  <0·001 |
| 180 days  P value | 0·823 (0·736-0·909)  ·· | 0·782 (0·693-0·871)  0·026 | 0·788 (0·700-0·876)  0·043 | 0·675 (0·580-0·771)  <0·001 | 0·681 (0·582-0·780)  <0·001 |
| 1 year  P value | 0·827 (0·743-0·911)  ·· | 0·788 (0·702-0·874)  0·027 | 0·788 (0·702-0·874)  0·044 | 0·678 (0·584-0·773)  <0·001 | 0·682 (0·584-0·779)  <0·001 |

P value of comparisons between COSSH-ACLF II score and the other scores. (z score test). LT, liver transplantation; HBV-ACLF, hepatitis B virus-related acute-on-chronic liver failure; COSSH-ACLF II score, Chinese Group on the Study of Severe Hepatitis B-ACLF II score; CLIF-C ACLF score, Chronic Liver Failure (CLIF)-Consortium ACLF score; MELD score, Model for End-Stage Liver Disease score; MELD-Na score, MELD-sodium score.
